# Microfluidic Liquid Cell with Silicon Nitride Super-Thin Membrane for Electron Microscopy of Samples in Liquid

**DOI:** 10.3390/bios12121138

**Published:** 2022-12-07

**Authors:** Akihiko Sugihara, Tadashi Ishida

**Affiliations:** Department of Mechanical Engineering, School of Engineering, Tokyo Institute of Technology, Kanagawa 226-8502, Japan

**Keywords:** super-thin electron transparent membrane, in-liquid scanning electron microscopy, liquid cell, PDMS microchannel, virus/bacteria

## Abstract

Microfluidic liquid cells have been developed to visualize nanoscaled biological samples in liquid using a scanning electron microscope (SEM) through an electron-transparent membrane (ETM). However, despite the combination of the high-resolution visualization of SEM and the high experimental capability of microfluidics, the image is unclear because of the scattering of the electron beam in the ETM. Thus, this study developed a microfluidic liquid cell with a super-thin ETM of thickness 10 nm. Because the super-thin ETM is excessively fragile, the bonding of a silicon–nitride-deposited substrate and a polydimethylsiloxane microchannel before silicon anisotropic etching was proposed prevented the super-thin ETM from damage and breakage due to etching. With this protection against etchant using the microchannel, the yield of the fabricated super-thin ETM increased from 0 to 87%. Further, the scattering of the electron beam was suppressed using a microfluidic liquid cell with a super-thin ETM, resulting in high-resolution visualization. In addition, T4 bacteriophages were visualized using a super-thin ETM in vacuum. Furthermore, the cyanobacterium *Synechocystis* sp. PCC6803 in liquid was visualized using a super-thin ETM, and sub-microscopic structures on the surface were observed.

## 1. Introduction

Infectious diseases are caused by viruses and bacteria, and the number of outbreaks has increased [1,2]. Recently, human beings have been affected by the coronavirus pandemic since 2019 (COVID-19) and have changed their way of life owing to the lack of sufficient countermeasures, such as vaccines and antivirus drugs for COVID-19. In bacterial infections, drug-resistant bacteria are a major issue [3]. To approach this issue, new mechanisms of action of antibiotics are required, which can be found in specific biological activities in bacteria [4]. Therefore, finding new viral and bacterial specific nanoscaled structures and their behaviors may lead to the development of antiviral and antibacterial action mechanism drugs. Although bright-field and florescent microscopy has found many viral and bacterial specific microstructures and their behaviors [5], fewer biological activities have been found recently due to the deflection limit. Thus, to find new viral- and bacterial-specific nanoscaled structures and their behaviors, a higher resolution is required to visualize viruses and bacteria in liquid conditions.

High-resolution imaging techniques often require fine probes, such as electron beam and sharp tip, for visualization. To visualize viruses and bacteria in liquid, the probes should be applicable in liquid and with less damage to viruses and bacteria. In situ imaging techniques for active viruses and living bacteria are preferred to remove pretreatments, such as fixation, dehydration, and staining, which inactivate viruses and kill bacteria, and affect the morphology, although the techniques usually need the pretreatments for high resolution imaging. In addition, resolution and frame rate are important. For the material analysis, the analysis and mapping capabilities of both organic and inorganic elements are important, because organic materials correspond to biological function and inorganic materials are essential for bacterial cultures. Furthermore, typical biological experiments require liquid control for biochemical reactions. These requirements are important for the visualization of viruses and bacteria in liquid.

High-resolution methods for visualizing in-liquid samples include atmospheric scanning electron microscopy (ASEM) [6], liquid cell transmission electron microscopy (TEM) [7], atomic force microscopy (AFM) in liquid [8], and super-resolution fluorescence microscopy [9]. ASEM has an electron-transparent membrane (ETM) between air for in-liquid samples and vacuum for electron beams. The ETM, which is usually silicon nitride (Si_3_N_4_), should be sufficiently thin for the transmission of the electron beam. With this ETM, in-liquid samples were visualized without pretreatment and mapped in terms of elemental and molecular distribution at a resolution of a few tens of nanometers. Furthermore, it sometimes combines with a fluorescent microscope for the distribution of specific organic materials [10]. Obtaining one image requires a few minutes. The combination of ETM and the microfluidic device enables the culture of biological samples and the performance of biological experiments with precise chemical control [11]. Liquid cell TEM also utilizes two ETMs, which sandwiches nanoscaled samples in-liquid. With high acceleration voltage, electron beams can transmit through the sandwiched in-liquid samples, resulting in in situ visualization and elemental mapping at sub-nm resolution. This high acceleration voltage can damage biological samples. Sometimes, it utilizes graphene, a single atomic layer of carbon, as the ETM and achieved sub-nm resolution at 3.85 fps [12], but there is low brightness due to its conductivity. However, to achieve the liquid cell TEM, the biological samples should be pre-treated, sliced, and thinned down to less than 1 μm, and therefore the experiments in such a tiny space are difficult. AFM can visualize the surface topology and force mapping of samples at the nanoscale, which also works in liquids [13]. It measures the frequency modulation of an AFM cantilever and scans the surface of the samples at a resolution of a few nanometers. With this, AFM does not need any pretreatments. The scan speed was increased, resulting in an image being acquired within a few seconds [14]. However, the fluid control sometimes affects the motion of the cantilever by fluid flow. A super-resolution fluorescent microscope can visualize the fluorescent molecular distribution at a higher resolution than the diffraction limit using data analysis or optical processes. There are different types of super-resolution fluorescent microscopy, such as stochastic optical reconstruction microscopy (STORM) [15] and stimulated emission depletion microscopy (STED) [16] which can achieve a resolution of a few tens of nanometers. They use fluorescence, which requires staining with fluorescent dyes. Obtaining one image requires a few minutes to complete. Similar to fluorescent microscopy, they can perform biological experiments.

Considering that the imaging techniques of living biological samples require high resolution, frame rate, less damage, no pretreatments, material analysis, and biological experiment, the ASEM is preferable. Although the ASEM fulfils all the requirements and resolution of a few tens of nanometers, the image resolution was not sufficiently high in the case of viruses and microstructures of bacteria. This is because the electron beam was scattered in the ETM. Thin ETM is better in terms of resolution. On the other hand, the ETM should be sufficiently strong not to be broken by the application of atmospheric pressure. The ETM should be thick in terms of strength. This trade off makes high resolution ASEM imaging difficult, resulting in a thick and narrow membrane structure (low resolution and narrow viewing area); for example, 100 nm in thickness and 250 μm × 250 μm in size for ASEM [17].

To improve the resolution of the ASEM and microfluidic liquid cell, the thickness of the ETM must be reduced while maintaining the viewing area. In this study, a microfluidic liquid cell with a super-thin ETM (~10 nm in thickness) of 100 μm × 100 μm in size was developed. Using this super-thin ETM, viruses in vacuum and nanostructures on bacteria in liquid can be visualized.

## 2. Microfluidic Liquid cell with a Super-Thin ETM

**Figure 1:**
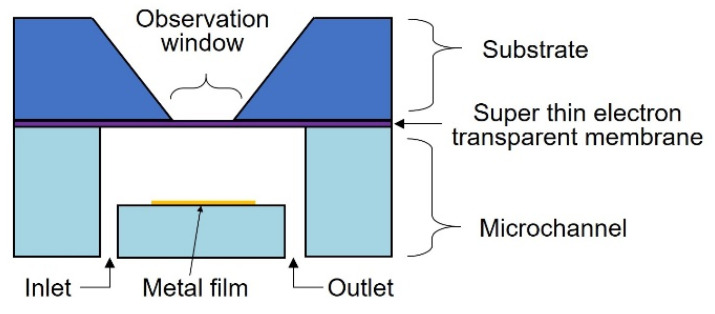
Schematic illustration of a microfluidic liquid cell with a super-thin ETM.

The microfluidic liquid cell with a super-thin ETM is shown in Figure 1. It consisted of a substrate with a super-thin ETM and a microchannel. The super-thin ETM covered the observation windows and suppressed the scattering of the electron beam within itself as much as possible; therefore, the probe size for SEM imaging did not expand significantly. The super-thin ETM was Si_3_N_4_ of 10 nm deposited by low-pressure chemical vapor deposition (LPCVD), which was thinner than the guaranteed foundry thickness (30 nm). The microchannel (SI) contained an inlet and outlet to introduce a solution for bacterial culture or cellular response to chemicals. The microchannel was composed of polydimethylsiloxane (PDMS). Owing to the gas permeability and optical transparency of PDMS, gas and visible light can be fed to the bacteria inside for bacterial culture. The bottom of the PDMS microchannel was locally coated with a Ti metal film to reduce charging during SEM imaging.

The PDMS microchannel and substrate with a super-thin ETM were bonded before wet etching of Si. The PDMS microchannel was chemically inert and worked as a protection of ETM against long-term wet etching even at ~10 Å/h etching rate [18]. It also protected stress caused by the flow of etchant in the step of ETM fabrication.

### 2.1. Design of Super Thin ETM

A super-thin ETM was designed in terms of electron beam scattering and strength. The scattering of the electron beam expands the probe size, which corresponds to the resolution. Smaller scattering is better. Consequently, strength was determined based on the size of the ETM. Sufficient strength was required to resist atmospheric pressure. Furthermore, a larger viewing area is preferable because more samples are visible through the ETM.

**Figure 2:**
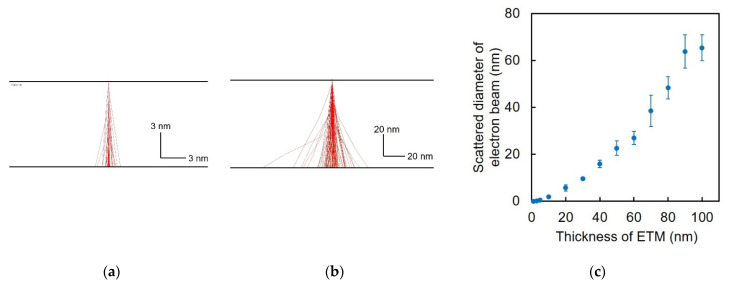
Monte Carlo simulation of electron beam scattering in a Si_3_N_4_ ETM. (**a**) Electron beam scattering in the Si_3_N_4_ membrane of 10 nm in thickness. (**b**) Electron beam scattering in the Si_3_N_4_ membrane of 80 nm in thickness. (**c**) Relationship between the thickness of the Si_3_N_4_ ETM and scattering diameter of electron beam at 17 kV in accelerating voltage.

To consider electron beam scattering, the electron beam trajectories were calculated using a Monte Carlo simulation. An electron beam of 17 keV was irradiated onto the ETM, with the ideal point spot as an input. Because the electron beams were scattered inside the ETM, the output spot size increased. Figure 2a,b show the scattering of the electron beam in the ETM of 10 nm and 80 nm, respectively. The calculated output spot size of the electron beam in the case of the 10-nm-thick Si_3_N_4_ membrane was 0.07 times compared to that in the case of the 80-nm-thick membrane (Figure 2c). The Monte Carlo simulation stochastically calculated 100 trajectories of the electron beam, and therefore, the averaged spot size and standard deviation were employed as error bars (N = 5 in trial). The probe size of SEM imaging was proportional to square of thickness of Si_3_N_4_. Therefore, the resolution dramatically increases when the ETM becomes thinner. Single Scattering Monte Carlo Simulation V 3.05 was used, with 22.46, 11.21, and 3.17 g/cm^3^ as effective atomic weight, effective atomic number, and density of Si_3_N_4_, respectively.

**Figure 3:**
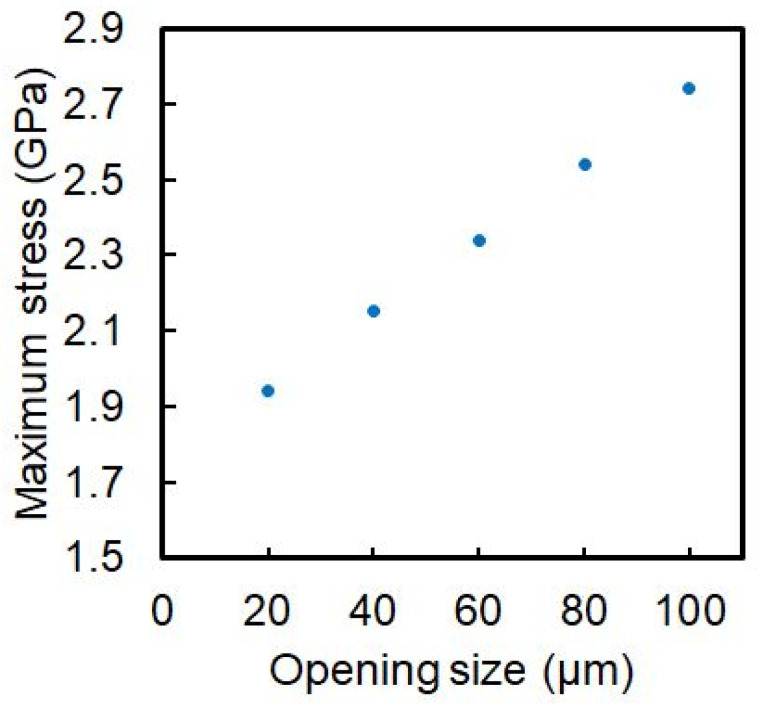
Maximum stress as a function of opening size in a 10-nm-thick Si_3_N_4_ membrane.

The maximum mechanical strength of the 10-nm-thick Si_3_N_4_ membrane was not high, while the maximum stress generated in the Si_3_N_4_ membrane dramatically increased in that range of thickness (SI and Figure S1). The strength is related to Young’s modulus, ETM thickness, shape, and size. Young’s modulus and ETM thickness and shape are decided due to the material, fabrication, and visualization point of view. To avoid breakage of the 10-nm-thick Si_3_N_4_ membrane owing to the application of atmospheric pressure, the size of the Si_3_N_4_ membrane should be adjusted. The maximum mechanical stress on a 10-nm-thick Si_3_N_4_ membrane was calculated by changing its size using the finite element method (FEM) (Figure 3). The fracture strength of Si_3_N_4_ has been reported to be in the range from 6 to 13 GPa [19,20]. The maximum stress must be lower than the fracture strength. In this case, to increase the fabrication yield of the super-thin ETM and to prevent the damage to SEM in case of the breakage of ETM, a safety ratio of 2 was set. The dimensions of the membrane were 100 μm × 100 μm. For the finite element method, COMSOL Multiphysics ver. 5.2a (COMSOL, Inc.) was used. Considering the symmetric shape of the model, the quarter FEM model was used for the simplification of the calculation. The model with two completely fixed boundaries and two boundaries fixed only in-plane motion and rotation. An equally distributed load was atmospheric pressure of 100 kPa, the density was 3100 kg/m^3^, Young’s modulus was 250 × 10^9^ Pa, and Poisson’s ratio was 0.23.

The observation window should be a tapered or shallow or large area to prevent the blockage of electrons. For the tapered shape, the anisotropic etching of silicon should be considered when designing the pattern of the observation windows. In the anisotropic etching of silicon, (111) is a crystalline facet that dramatically reduces the etching rate compared to (100) and (110) [21]. The angle between the (100) and (111) planes was 54.7°. When the length of one side of the observation window, α, is 100 μm, the length of the pattern on the opposite side of the silicon substrate, β, should be designed according to Equation (1), where t is the thickness of the silicon substrate.

(1) $$\alpha =\beta \;-\frac{2t}{\;\tan54.7{}^{\circ}},$$

### 2.2. Fabrication Process

The ETM was fabricated by etching Si with tetramethylammonium hydroxide (TMAH), which also etched and damaged Si_3_N_4_ at ~10 Å/h [18]. The damages to the ETM were on the several nm scale due to long etching time, and strongly affected the strength of the super-thin ETM. This is because its thickness was only 10 nm, which is not large in comparison to the damage. To avoid this damage, the pre-bonding of a silicon substrate with Si_3_N_4_ layers and a microchannel before the etching of silicon were proposed in this study. The bonded microchannel functioned as a protective layer of Si_3_N_4_ against etching and damages caused by the flow of liquid and mechanical contacts.

**Figure 4:**
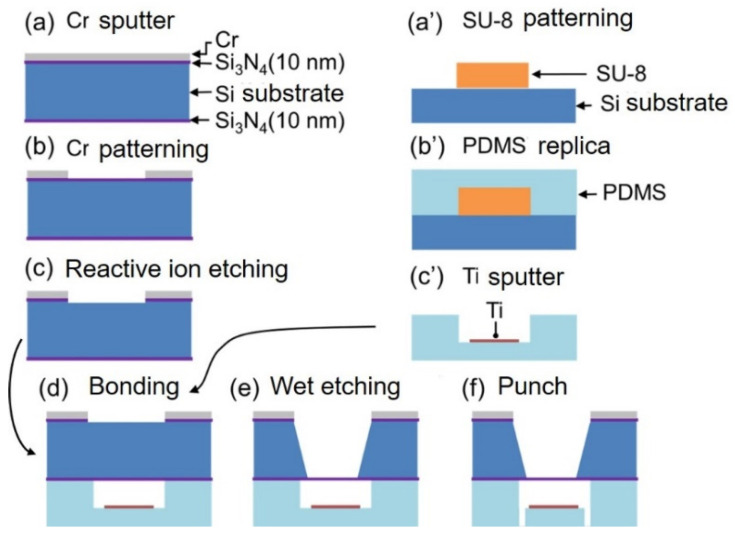
Fabrication process of the microfluidic liquid cell with the super-thin ETM.

Figure 4 shows the fabrication process of a microfluidic liquid cell with a super-thin ETM. A Cr layer with a thickness of 150 nm was sputtered on a silicon substrate ((100), 300 nm thickness) with a Si_3_N_4_ thickness of 10 nm deposited by LPCVD (Figure 4a). The Cr layer was patterned as a mask for etching (Figure 4b). Reactive ion etching of SF_6_ was performed to locally etch the Si_3_N_4_ membrane to etch Si (Figure 4c). A PDMS microchannel was prepared by soft lithography, an SU-8 mold was fabricated by photolithography and should be treated by hexamethyldisilazane (HMDS) vapor treatment for the detachment of PDMS replica (10 min exposure to HMDS vapor and 1 min baking at 95 °C) (Figure 4a’). PDMS (base to agent was 10:1, cure at 100 °C for 60 min) was cast on the SU-8 mold. A PDMS replica was fabricated (Figure 4b’). Ti with a thickness of 100 nm was locally sputtered onto the PDMS microchannel through a shadow mask hole (Figure 4c’). Further, the patterned substrate and PDMS microchannel were bonded by irradiation with vacuum ultraviolet light (Figure 4d). The bonded structure was anisotropically etched with TMAH at 90 °C until a freestanding super-thin ETM was formed (about 4 h 20 min) (Figure 4e). The inlet and outlet of the microfluidic liquid cell were formed using a biopsy punch (Figure 4f). Without any additional processes, microfluidic liquid cell is available.

### 2.3. Experimental Setup

**Figure 5:**
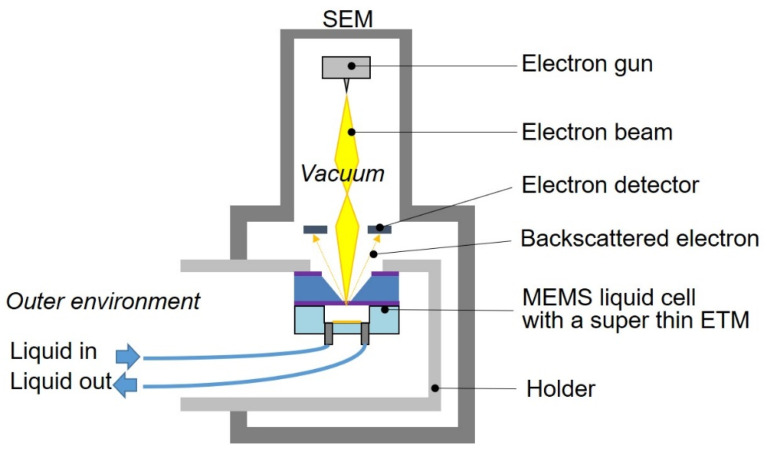
Experimental setup of the microfluidic liquid cell with a super-thin ETM in SEM.

Figure 5 shows the experimental setup using the microfluidic liquid cell. The microfluidic liquid cell with the super-thin ETM was placed and glued onto a special specimen holder with an epoxy resin. The specimen holder was installed in a SEM (BiSEM-10, Technex Lab Co., Ltd., Tokyo, Japan) and connected to the outer environment. Thereafter, the super-thin ETM was separated into air and vacuum, where almost 100 kPa was applied. The microchannel was located on the side of the air and was connected to a syringe pump for the introduction of cyanobacterial suspensions and the perfusion of the culture medium. To bring cyanobacteria close to the super-thin ETM, a cyanobacterial suspension was introduced with a flow rate of 2 μL/min, stopped, and then the substrate was placed down for 30 min.

When only a super-thin ETM without a microchannel was used for the characterization, the suspension containing gold nanoparticles or T4 bacteriophages was dropped and dried on the super-thin ETM. The dried samples prepared on the super-thin ETM were introduced into the SEM specimen chamber for SEM imaging. On the side of the vacuum, it was 10^−3^ Pa for the generation and irradiation of the electron beam. The acceleration voltage was 17 kV and the spatial resolution was 10 nm. In addition, backscattered electrons were used for visualization rather than secondary electrons in terms of the electron energy to pass through the super-thin ETM.

## 3. Methods and Materials

### 3.1. Samples for SEM Observation

The suspension of gold nanoparticles was purchased from Sigma Aldrich. The diameter was 200 ± 20 nm, and the density was 1.9 × 10^9^ particles/mL. T4 bacteriophage is a virus that infects *Escherichia coli* [22]. The size was approximately 200 nm. It was amplified by infection with *Escherichia coli* at 37 °C and collected from the suspension. The density was 1.0 × 10^10^–1.0 × 10^11^ pfu/mL. *Synechocystis* sp. PCC6803, is a cyanobacterium [23]. The size was 2–3 μm in diameter. The cells were cultured on BG11 agar in an incubator at 34 °C with fluorescent lamps.

### 3.2. Method to Check Existence of Super-Thin ETM

The super-thin ETM was too thin to confirm its existence using total reflection of light. The thickness of the super-thin ETM was measured on Si substrates before the fabrication process by a spectroscopic film thickness measurement system (Nanospec, Nanometrics Inc.). To check the existence of the super-thin ETM, a laser marker of a profile micrometer (VF-7510, Keyence) was used. When the laser marker is focused on a flat surface, the shape of the marker is linear. If the surface had bumps and dips, the marker was defocused and disconnected at its location. Using these characteristics, when a super-thin ETM existed, a line-shaped laser marker could be observed in the observation window. When it did not exist, disconnection in the line-shaped laser marker was observed.

### 3.3. Method to Measure Diameter of Gold Nanoparticles

The diameters of gold nanoparticles could not be easily determined, because the SEM images of the gold nanoparticles did not show clear edges. The HWFM was used as an indicator of the size of a gold nanoparticle. The intensity profile along a line crossing the center of a nanoparticle is extracted from the SEM image. The profile of a nanoparticle always has one peak, and the HWFM can be measured. The HWFMs of three nanoparticles were averaged. Image analyses were performed using the ImageJ software (NIH).

## 4. Results

### 4.1. Fabricated Device

**Figure 6:**
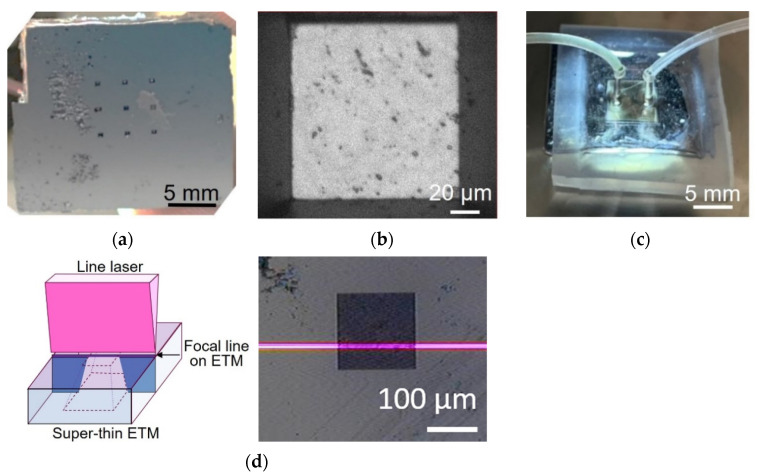
Fabricated microfluidic liquid cell with the super-thin Si_3_N_4_ membrane. (**a**) Entire microfluidic liquid cell from the substrate side. (**b**) Close-up image of observation window. (**c**) Entire microfluidic liquid cell from the microchannel side. (**d**) Focused line laser was projected on flat super-thin ETM if the super-thin ETM existed. Focused line laser was not projected if the super-thin ETM was broken. Laser marker was visible on the super-thin ETM.

Figure 6 shows the fabricated microfluidic liquid cell with a super-thin ETM. The thickness of the Si_3_N_4_ membrane was 10.8 +/− 1.1 nm (N = 3 in substrate, error was standard deviation). Nine observation windows with dimensions of 100 μm × 100 μm were located on the side of the substrate (Figure 6a). Inside the observation windows, there was a super-thin ETM, although it was not visible (Figure 6b). The microchannel was aligned over the observation windows (Figure 6c). Further, the existence of the super-thin ETM was confirmed using a laser profiler (Figure 6d). The fabrication yield rate of the super-thin ETM using the proposed fabrication process was 87%, whereas that obtained using the conventional fabrication process, that is, isotropic wet etching of the silicon substrate before bonding, was 0%.

The super-thin ETM was very fragile; therefore, it was checked whether the liquid introduction and pressure application broke the super-thin ETM. When the ETM was broken, the observation window was completely open due to its tensile tension. To confirm that the introduction of liquid did not break the super-thin ETM, water was not perfused from the inlet to the observation window, but to the outlet (Figures S2 and S3). The flow rate was 2–10 μL/min, and the duration was 1 h. The super-thin ETMs were not broken, and water exited only from the outlet. The microfluidic liquid cell with the super-thin ETM was installed into SEM. Atmospheric pressure was applied to the super-thin ETM and they were found to not be broken for 24 h. If the ETM was broken, electron beams cannot be generated, resulting in no SEM images. Thanks to the ETM, electron beams could visualize the tapered observation window using backscattered electrons (Figure S4).

The thinner ETMs were developed for TEM observation. A free-standing Al_2_O_3_ membrane deposited by atomic layer deposition, which was 5 nm in thickness and 20 μm × 5 μm in size, was achieved [24], but did not withstand atmospheric pressure due to lower fracture strength. It required 15 nm in thickness to withstand the pressure, even though the viewing area was small. The free-standing graphene membrane, whose thickness was 0.35 nm, only sandwiched nanoscaled specimens between the layers for observation [12]. The observation area, where specimens were sandwiched, was small. With this, the integration of the graphene membrane and microfluidic devices was difficult. If a thinner super-thin Si_3_N_4_ membrane can be deposited, microfluidic liquid cells with the super-thin Si_3_N_4_ membrane will achieve high resolution imaging, large viewing area, and high experimental capability in comparison to other ETMs.

### 4.2. SEM Observation Using the Device

#### 4.2.1. Gold Nanoparticle

**Figure 7:**
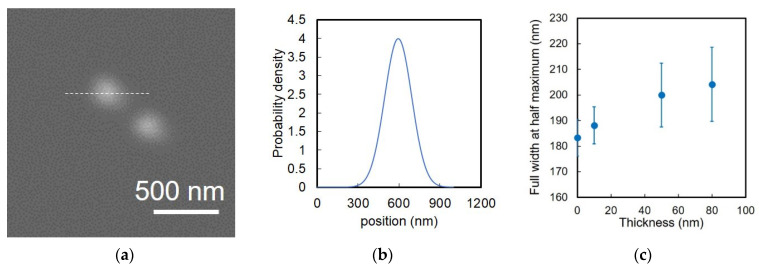
Full width at half maximum of gold nanoparticles of 200 nm in diameter. (**a**) SEM image of gold nanoparticles. (**b**) Brightness distribution along white dash line over a gold nanoparticle in (**a**). (**c**) Full width at half maximum of gold nanoparticles as a function of thickness. Error bar was standard deviation of diameter of three gold nanoparticles.

Figure 7 is the measurement of diameter of gold nanoparticles with diameters of 200 nm in SEM images. A suspension of gold nanoparticles was dropped and dried on a super-thin ETM. The gold nanoparticles were observed on or through the super-thin ETMs of different thicknesses (Figure S5). Figure 7a was the SEM image of the gold nanoparticles. The diameters of the gold nanoparticles were measured by the half-width at full maximum (HWFM) of brightness distribution along a line across the gold nanoparticles in SEM images and calculated probability densities; assuming the distribution is Gaussian (Figure 7b and Figure S5), Si_3_N_4_ films of 10, 50, and 80 nm in thickness were used as ETMs. The gold nanoparticles were visualized through the ETMs and the HWFMs were measured and plotted in Figure 7c. Note that 0 nm thick ETM was the case of gold nanoparticles on the ETM and direct exposure to electron beams. The HWFM in the case of direct SEM image of the gold nanoparticles on the ETM was 183 +/− 7.2 nm, while the diameter was 200 ± 20 nm on the specification sheet. While the HWFM is a good indicator to decide the width of brightness distribution with Gaussian curve in images, it is not the exact diameter. This is because it loses its outer side of brightness distribution, resulting in a smaller diameter than the exact one. As expected, the HWFM increased when the ETM increased due to the electron beam scattering. According to Figure 7c, the HWFM difference was 5 nm between the ETM of 0 and 10 nm in thickness. This was one-fourth of that between 0 and 80 nm (20 nm). With this improvement, the HWFM difference with/without the super-thin ETM was less than 10 nm. This meant that the shapes of sub-μm-scaled structures could be visualized in the atmospheric or in-liquid environments.

#### 4.2.2. Bacteriophage

**Figure 8:**
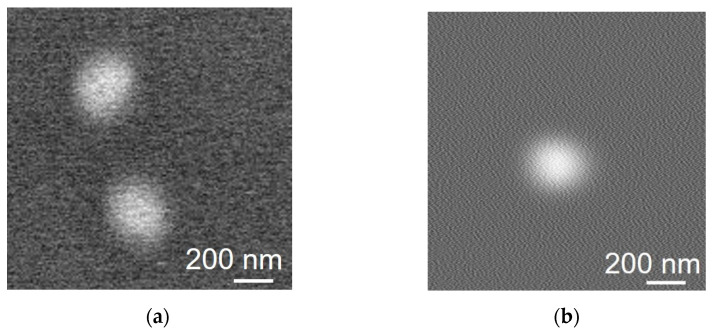
SEM images of T4 bacteriophage through the super thin ETM. (**a**) T4 bacteriophage on the ETM. (**b**) T4 bacteriophage through the super thin ETM.

Figure 8 shows the SEM images of the T4 bacteriophages. A suspension of T4 bacteriophages was dropped and dried on a silicon substrate with a Si_3_N_4_ layer or a super-thin ETM. The SEM images were obtained from the other side of the super-thin ETM. Figure 8a shows an SEM image of the T4 bacteriophage on the ETM. Figure 8b shows an SEM image of the T4 bacteriophage through the super-thin ETM. To compare the size of the bacteriophages between them, we used averaged FWMHs between short and long diameters. The FWMH of the direct SEM image of the bacteriophages on the ETM was 230 nm, and through the super-thin ETM was 248 nm. Although they displayed a 7.8% difference, this amount of difference was acceptable because of the variation and orientation of biological samples. Therefore, both images appeared similar and applicable.

#### 4.2.3. Cyanobacteria in Liquid

**Figure 9:**
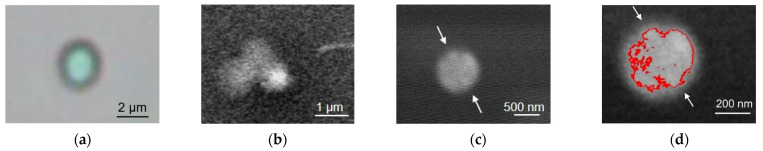
Images of cyanobacteria in liquid using the microfluidic liquid cell with the super-thin ETM. (**a**) Bright-field optical microscope image of cyanobacteria in liquid. (**b**) SEM image of bacterium in liquid through an 80-nm-thick ETM. (**c**) SEM image of bacterium in liquid through a super-thin ETM. It has microstructures (white arrows). (**d**) Outline of bacterium in processed SEM image.

Figure 9 shows images of the cyanobacteria *Synechocystis* sp. PCC6803 in the culture medium. Figure 9a shows an optical microscopy image. The cyanobacteria appeared elliptical, which may indicate an early stage of cell division. Figure 9b,c show SEM images of the cyanobacteria through the 80-nm-thick ETM and the super-thin ETM, respectively. In the case of the 80-nm-thick ETM, the aggregated cyanobacteria were blurred, and it is difficult to identify their microstructures at the 100 nm scale. In the case of the super-thin ETM, the cyanobacterium was circular with tiny sub-micrometer structures (arrows in Figure 9c). To confirm the existence of the sub-micrometer structures, the SEM image was processed in the typical method, that is, Gaussian blur filter and image thresholding (Figure 9d). According to the outline, the structures were clearly observed.

From the comparison between the bright-field optical microscope image, SEM images through 80-nm-thick and super-thin ETMs, the 100-nm-scaled microscopic structures of the cyanobacterium can be seen through the super-thin ETM, although it is difficult to observe those in the bright-field optical microscope image and the SEM image through the 80-nm-thick ETM. The diameter was small in SEM images; the 80-nm-thick or super-thin ETM were approximately 1 μm and 500 nm, respectively. This may imply that only parts of the cyanobacterium close to the ETMs were visible with small scattering of the electron beam, whereas those far from the ETMs were invisible because of large scattering by the culture medium.

## 5. Conclusions

This study developed a microfluidic liquid cell with a thin ETM. The super-thin ETM was fabricated via anisotropic wet etching of silicon bonded to a PDMS microchannel. The PDMS microchannel prevented etching of the super-thin ETM. Through the super-thin ETM, gold nanoparticles, bacteriophages, and cyanobacteria in the liquid were visible. Microstructures of biological samples at the 100 nm scale could be observed. For better imaging resolution, the scattering in liquid should be suppressed by adjusting the distance between the super-thin ETM and nanoparticles. In the future, using a super-thin ETM, the dynamics of viruses and bacteria will be observed in situ during biological experiments, in which the microfluidics will control physical and chemical inputs precisely, and induce the biological reactions. The obtained information is important for the biological study of, and drug discovery against, viruses and bacteria.

## Data Availability

Not applicable.

## Supplementary Materials

The following supporting information can be downloaded at: https://www.mdpi.com/article/10.3390/bios12121138/s1, Figure S1: Maximum stress as a function of thickness of ETM; Figure S2: Leakage test of water; Figure S3: Leakage water from an observation window with a broken ETM; Figure S4: Backscattered electron image of an observation window with a suspending super-thin ETM; Figure S5: SEM images of gold nanoparticles through the ETM.
