# Supplementary material for: Microfluidic Liquid Cell with Silicon Nitride Super-Thin Membrane for Electron Microscopy of Samples in Liquid
Source: Biosensors (Basel). 2022 Dec 7;12(12):1138. doi: 10.3390/bios12121138 (PMC9775586; doi:10.3390/bios12121138)
Supplement: Supplementary file 1 [file biosensors-12-01138-s001.zip › biosensors-1994837-supplementary.pdf]

Supporting materials

Microfluidic Liquid Cell with Silicon Nitride Super-Thin Membrane for Electron Microscopy of Samples in Liquid

Akihiko Sughihara and Tadashi Ishida *

Dimensions of microchannel

The microchannel was for the protection layer in this study, and therefore, it was designed to cover 9 observation windows. For this purpose, the microchannel was wide. The width was 10 mm, the length was 10 mm, and the height was 30 µm.

Maximum stress applied to ETM

The maximum stress was plotted as a function of thickness of ETM with the following equations considering the ETM was circular for the simplification (Fig. R1).

$$\frac{\delta}{t} + 0.471\left(\frac{\delta}{t}\right)^3 = 0.171\frac{p}{E}\left(\frac{r}{t}\right)^4 ,$$

$$\sigma = 0.476\mathrm{E}\left(\frac{\delta}{r}\right)^2 + 4.40\mathrm{E}\frac{t\delta}{r^2},$$

where δ is the deflection at the center, r is radius, t is thickness, p is pressure, E is Young's modules, σ is maximum stress. Even though 20-nm thick ETM (thin ETM available in commercial products) was achieved using conventional fabrication method, the maximum stress dramatically increased when the ETM became thin. The maximum stress at 10-nm thick ETM was 1.6 times higher than that at 20-nm ETM, resulting in easy breakage.

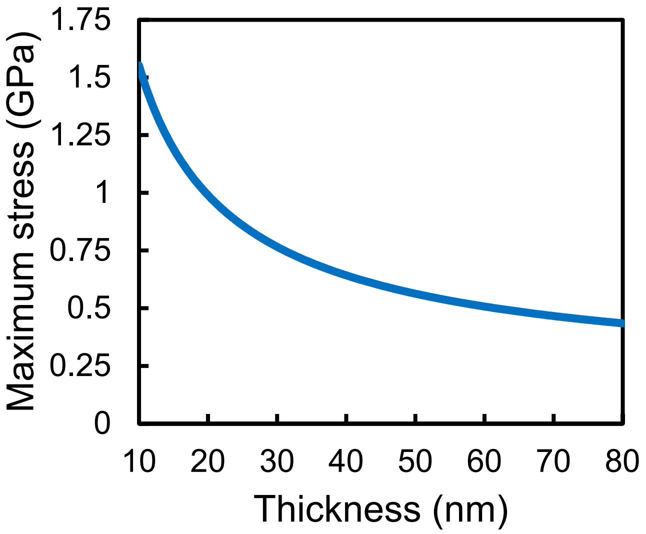

Figure S1. Maximum stress as a function of thickness of ETM.

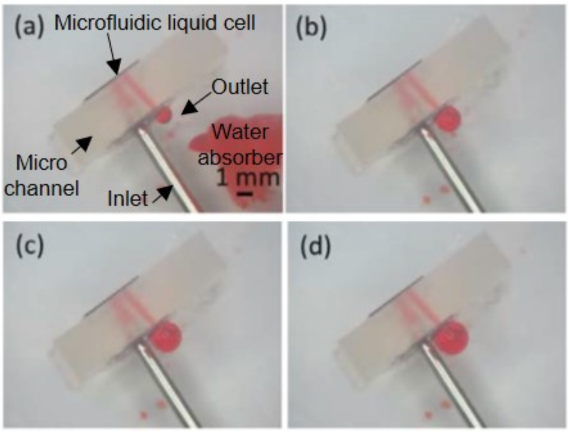

Figure S2. Leakage test of water. Red-dyed water was introduced into the microfluidic liquid cell, and the water did not come out from an observation window, but from the outlet. The observation window was sealed with ETM without breakage (a) 0 s. (b) 4 s. (c) 8 s. (d) 12 s.

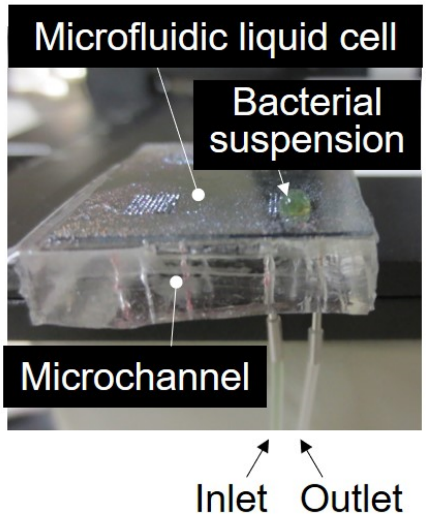

Figure S3. Leakage of water from an observation windows with a broken ETM. Suspension of cyanobacteria was introduced into the microfluidic liquid cell from an inlet, and the suspension came out from observation windows where ETM was broken.

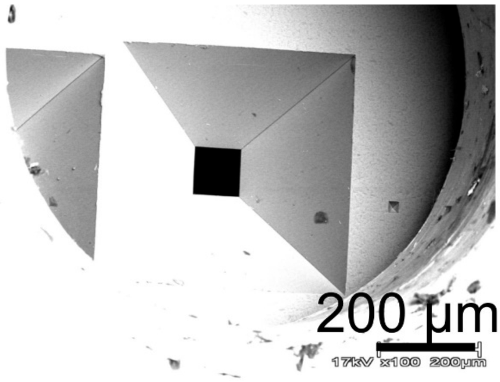

Figure S4. Backscattered electron image of an observation window with a suspending super-thin ETM.

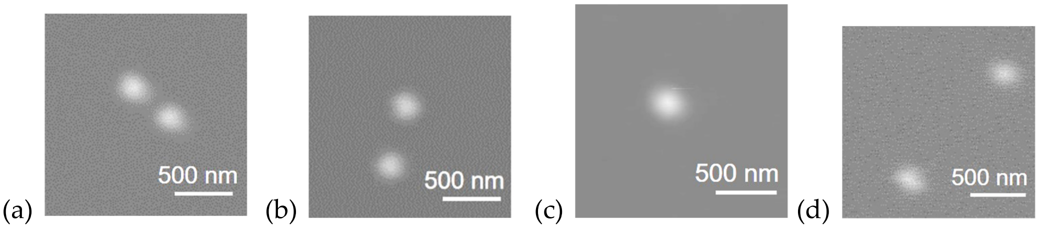

Figure S5. SEM images of gold nanoparticles through the ETM. (a) direct SEM imaging of gold nanoparticles on a super-thin ETM. (b-d) SEM imaging of gold nanoparticles through super-thin ETMs of 10, 50, 80 nm in thickness, respectively.
